# Prevalence of Portal Hypertensive Gastropathy in Chronic Liver Disease and Correlation with the Severity of Liver disease

**DOI:** 10.7759/cureus.5454

**Published:** 2019-08-21

**Authors:** Pratap S Tiwari, Sudhamshu KC, Dilip Sharma, Mukesh S Paudel, Amrendra Mandal

**Affiliations:** 1 Hepatology, National Academy of Medical Sciences, Kathmandu, NPL; 2 Gastroenterology, Lumbini Medical College, Palpa, NPL; 3 Internal Medicine, Interfaith Medical Center, Brooklyn, USA

**Keywords:** chronic liver disease, cirrhosis, portal hypertensive gastropathy, esophageal varices, congestive gastropathy

## Abstract

Background

Portal hypertensive gastropathy (PHG) is an underappreciated condition in patients with chronic liver disease (CLD). It is a common endoscopic finding in CLD patients, but its relation with esophageal varices (EV) and the severity of the liver disease is controversial. Herein, we aimed to study the prevalence of PHG in CLD patients and to determine its association with EV and the severity of the liver disease.

Methods

This descriptive, cross-sectional, analytical study was conducted at the Hepatology department, Bir Hospital Kathmandu from 19^th^ March to 30^th^ June 2019. A total of 404 patients with CLD of various etiology fulfilling the inclusion criteria were approached, and informed consent was taken before enrolling in the study. All patients underwent EGD, and the findings related to EV and PHG were noted. The severity of PHG was graded according to the McCormack classification and EV were graded according to the American Association for the study of liver diseases guideline. The severity of liver disease was stratified based on Child-Pugh class and Model for End-Stage Liver Disease (MELD score). Data was entered on Statistical Package for the Social Sciences (SPSS) Version 25 for further analysis.

Results

Of 404 CLD patients, the mean (±SD) age was 49.14 (±10.5) years. Portal hypertensive gastropathy was observed in 269 (66.6%) patients, of which 80.6% (217) had mild PHG while 19.4% (52) had severe PHG. EV were present in 362 (89.6%) patients. One hundred and thirty-two (36.5%) had small EV, and 230 (63.5%) had large EV. No significant association was observed between grades of gastropathy and size of varices (*p *= 0.36). There was a non-significant association with the MELD score and other biochemical parameters. However, there were significant associations between Child-Pugh class and PHG and Child-Pugh class and PHG severity, *p *= 0.001 and *p *= 0.01 (*p* <0.05), respectively.

Conclusions

In our study, the prevalence of PHG in the Nepalese population in CLD is 66.6 %. PHG is significantly associated with the severity of CLD in terms of Child-Pugh class but not associated with MELD. Also, no association has been found with the size of varices.

## Introduction

The term “Portal hypertensive gastropathy” is used to define the characteristic appearance which is a mosaic-like pattern or a diffuse, erythematous and reticular cobblestone pattern of gastric mucosa consisting of small polygonal areas, with or without superimposed red punctate lesions, >2 mm in diameter and a depressed white border [1-3]. Portal hypertensive gastropathy (PHG) is diagnosed based on esophagogastroduodenoscopy (EGD) findings [4]. 

Endoscopic classification of PHG severity is clinically crucial because severity is correlated with bleeding risk with an increased risk of gastric hemorrhage in severe (38% to 62%) compared with mild cases (3.5% to 31%) [5-8]. McCormack et al. classified PHG as “Mild” with features like fine pink speckling (scarlatina-type rash), and mosaic pattern (snakeskin appearance) and “Severe” as discrete red spots or diffuse hemorrhagic lesion [9]. 

Several studies have been carried out in different population groups around the world to find its prevalence that varies significantly from 16% to 100% in patients with chronic liver disease (CLD) [10]. There is a paucity of literature regarding PHG in the Nepalese population with CLD. The wide variation in the reported prevalence is perhaps related to patient selection, absence of uniform criteria and classification, and more importantly, the differences in interobserver variation [11-12]. 

This study aimed to find out the prevalence of PHG in CLD patients in the Nepalese population and to see the association of PHG with the severity of the liver disease.

## Materials and methods

Recruitment of participants

This was a descriptive, cross-sectional, analytical study conducted at the National Academy of Medical Sciences, Bir Hospital, Kathmandu, Nepal between 19^th^ March and 30^th^ June 2019. Informed consent for participation was obtained from all the participants. Consecutive patients of CLD attending Hepatology unit, irrespective of etiology, diagnosed during the study period, were enrolled in the study. Online sample size calculator was used using the prevalence of disease as 50 %, and the calculated size was 384 [13]. CLD was diagnosed on the basis of history, clinical examination, laboratory parameters, imaging diagnosis, and/or a histopathological examination (if necessary). Patient unwilling to give consent, or with active upper gastrointestinal bleed, or with ongoing comorbid conditions like acute exacerbation of chronic obstructive pulmonary disease/asthma, myocardial infarction (within six months), and patients on the ventilator were excluded. Patients having Hepatocellular carcinoma, portal vein or splenic vein thrombosis, Severe alcoholic hepatitis, acute on chronic liver failure, non-cirrhotic portal hypertension, and CLD of unknown etiology/mixed etiology were excluded. Patients on beta-blockers, Non-steroidal anti-inflammatory drugs, proton pump inhibitors, and active bleeding were also excluded. All the patients underwent EGD, under the guidance of endoscopist (>15 years of experience). Findings suggestive of PHGwere noted and graded as per the McCormack criteria [9]. The presence of esophageal varices (EV) was noted and graded as small varices (straight, <5 mm) and large EV (tortuous >5 mm) as per the American Association for the Study of Liver Disease Guidelines [14].

Stratifying liver disease severity

Complete blood count, renal function test, liver function test, abdominal ultrasonography, prothrombin time, INR level data were collected. The severity of liver disease was assessed by Child-Pugh class and model for end-stage liver disease (MELD) score [15-16]. We stratified CLD patients into three groups i.e., MELD <10, MELD 10-15 and MELD >15.

Statistical analysis

Continuous variables were expressed as mean (±SD) and discrete variables as numbers and percentage. Continuous variables were compared by using Student T-test or Mann Whitney as relevant and discrete variables by chi-square test or Fischer’s exact test as relevant. Pearson’s correlation coefficient assessed bivariate correlation. Statistical Package for the Social Sciences (SPSS) version 25 was used for statistical analysis. A two-sided *p*-value of <0.05 was considered significant.

## Results

A total of 468 patients were diagnosed to have CLD during the study period. Sixty-four patients were excluded because of non-cirrhotic portal hypertension (six), alcoholic hepatitis (14), acute on chronic liver failure (six), CLD of mixed and uncertain etiology (25), beta-blockers (four), proton pump inhibitors (six), and the presence of active bleeding (three). Subsequently, 404 patients were subjected to EGD examination after initial evaluation. The patients’ general characteristics are summarized in Tables 1 and 2.

**Table 1:** Demographic profile of the study population PHG, portal hypertensive gastropathy

Variables		No PHG	PHG		p-value
			Mild	Severe	
Gender, n (%)	Male	101 (25.0)	153 (37.9)	40 (9.9)	0.51
	Female	34 (8.4)	64 (15.8)	12 (3.0)	
Total: 404		135 (33.4)	217 (53.7)	52 (12.9)	

**Table 2:** Laboratory parameters of the study population PHG, portal hypertensive gastropathy, AST, aspartate aminotransferase; ALT, alanine aminotransferase; INR, international normalized ratio, MELD, model for end-stage liver disease; SD, standard deviation

Parameters	No PHG (n = 135)	PHG (n = 269)	p-value
Hemoglobin, Mean gm/dl (±SD)	9.74 (1.93)	9.76 (1.88)	0.89
Platelet, X10^9^/L	123.83 (51.54)	125.26 (44.24)	0.78
AST, IU/L	89.00 (83)	100 (88)	0.13
ALT, IU/L	40.00 (23)	44 (30.5)	0.57
Bilirubin, mg/dl	2.50 (4.7)	3.00 (4.1)	0.89
Albumin, g/dl	2.7 (0.8)	2.8 (0.7)	0.55
INR	1.58 (0.45)	1.59 (0.45)	0.78
Creatinine, mg/dl	1.07 (0.56)	1.02 (0.53)	0.35
MELD score	16.63 (6.24)	16.99 (6.19)	0.58

**Figure 1:**
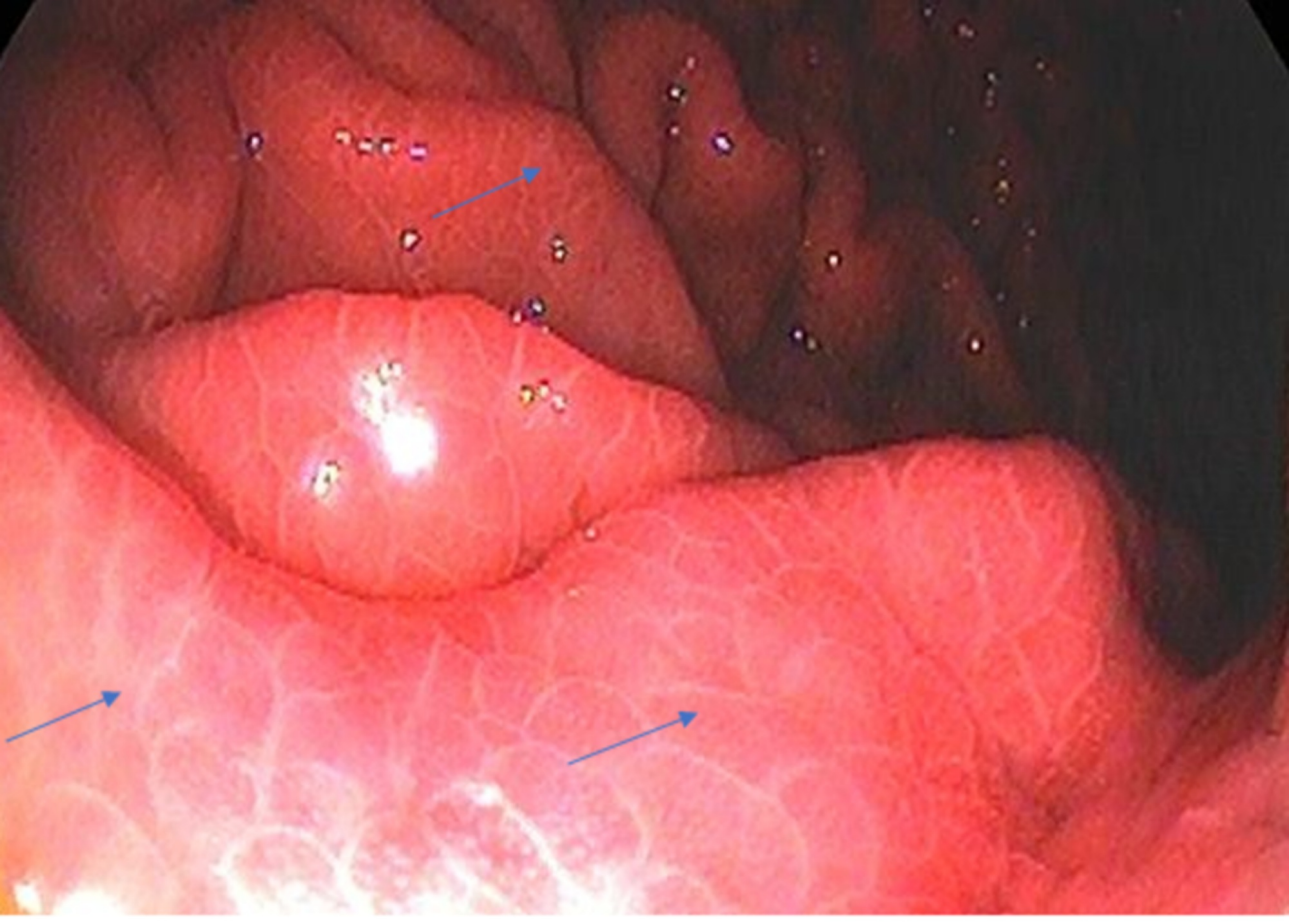
Mild PHG showing reticular cobblestone pattern of gastric mucosa (blue arrows) PHG, portal hypertensive gastropathy

**Figure 2:**
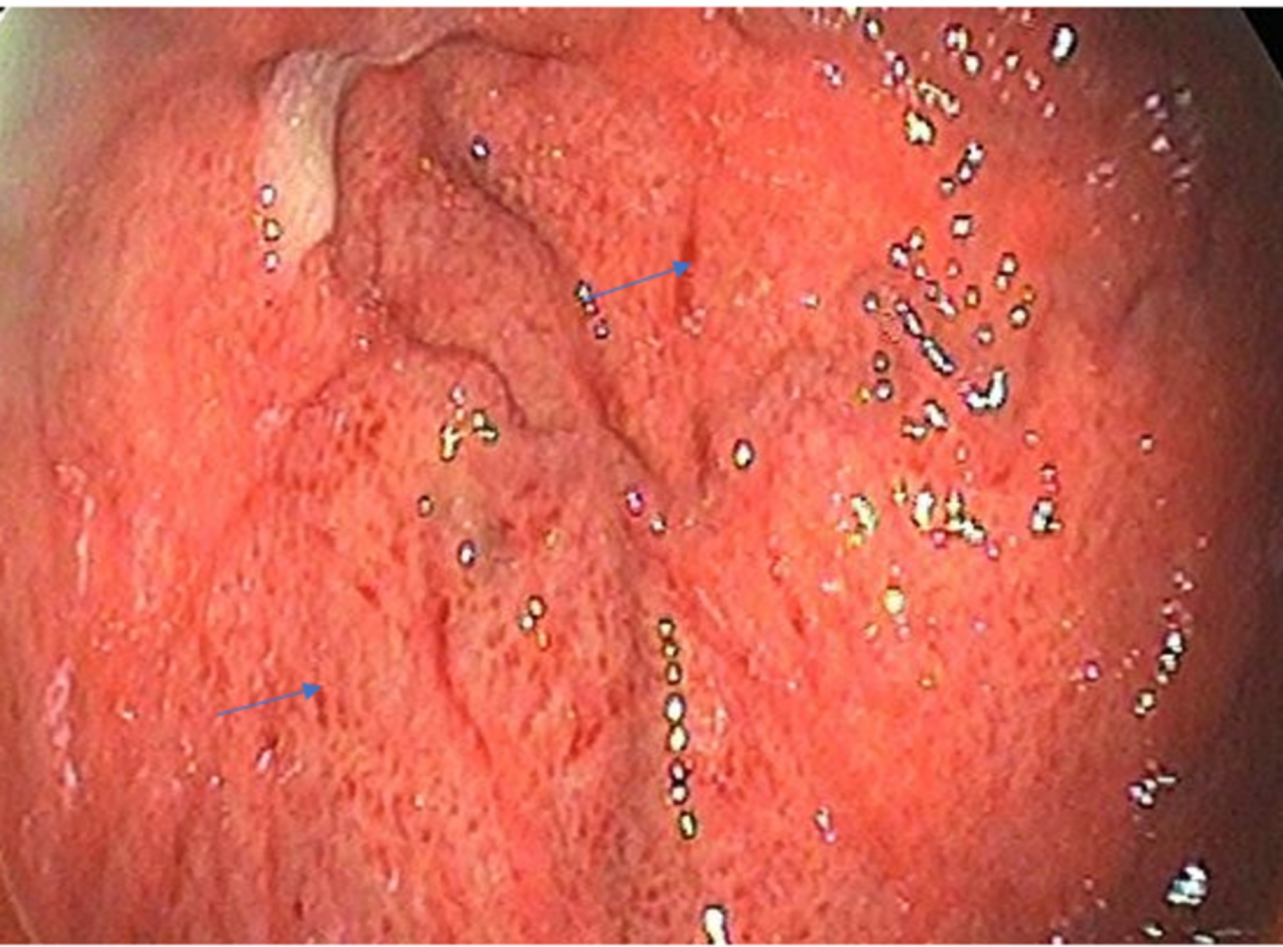
Severe PHG as discrete red spots and diffuse hemorrhagic lesion (blue arrows) PHG, portal hypertensive gastropathy

In EGD examination, 269 (66.6%) patients had PHG, of which 80.6% (217) had mild PHG (Figure 1), while 19.4% (52) had severe PHG (Figure 2).

The difference in the distribution of PHG between male and female was not significant.

Factors related to PHG and EV

Of total patients, EV was present in 362 (89.6%) patients, whereas PHG was present in 243 (67.1%) patients. Sub-group analysis was done among the 362 patients with EV. One hundred and thirty-two (36.5%) had small EV, and 230 (63.5%) had large EV. Among the patients with small varices, mild PHG and severe PHG was present in 70 (19.3%) and 22 (6.1%) patients respectively, and PHG was absent in 40 (11.0%) patients. Similarly, among patients with large varices, mild PHG and severe PHG was present in 128 (35.4%) and 23 (6.4%) patients respectively, and PHG was absent in 79 (21.8%) patients. The non-significant association was found between PHG and EV (*p *= 0.49), as shown in Tables 3 and 4.

**Table 3:** Distribution of varices among CLD patients PHG, portal hypertensive gastropathy; EGD, esophagogastroduodenoscopy; CLD, chronic liver disease

EGD findings	PHG		Total n (%)
	No PHG n (%)	PHG n (%)	
Varices present	119 (88.1)	243 (90.3)	362 (89.6)
No Varices	16 (11.9)	26 (9.7)	42 (10.4)
Total	135 (100)	269 (100)	404 (100)

**Table 4:** Portal hypertensive gastropathy severity in relation to various grades of esophageal varices PHG, portal hypertensive gastropathy

		PHG			Total
		None n (%)	Mild n(%)	Severe n(%)	
Esophageal varices	Small	40 (11.0)	70 (19.3)	22 (6.1)	132 (36.5)
	Large	79 (21.8)	128 (35.4)	23 (6.4)	230 (63.5)
Total		119 (32.9)	198 (54.7)	45(12.4)	362 (100)

Factors related to PHG: liver disease severity

Liver disease severity was assessed by Child-Pugh class and MELD score. Fifty (12.4%) patients were in Child-Pugh class A, 142 (35.1%) in Child-Pugh class B and 212 (52.5%) patients were in Child-Pugh class C. Patients “without PHG’ and “with PHG” were 32 (7.9%) and 18 (4.5%) in Child-Pugh class A, 36 (8.9%) and 106 (26.2%) in Child-Pugh class B and 67 (16.6%) and 145 (35.9%) in Child-Pugh class C, respectively, as shown in table 5.

**Table 5:** Distribution of portal hypertensive gastropathy according to Child-Pugh class among CLD patients PHG, portal hypertensive gastropathy; CLD, chronic liver disease

	PHG		TOTAL
	No PHG n (%)	PHG n (%)	
Child-Pugh class A (CPS <7)	32 (7.9)	18 (4.5)	50 (12.4)
Child-Pugh class B (CPS 7-9)	36 (8.9)	106 (26.2)	142 (35.1)
Child-Pugh class C (CPS >9)	67 (16.6)	145 (35.9)	212 (52.5)
Total	135 (33.4)	269 (66.6)	404

**Figure 3:**
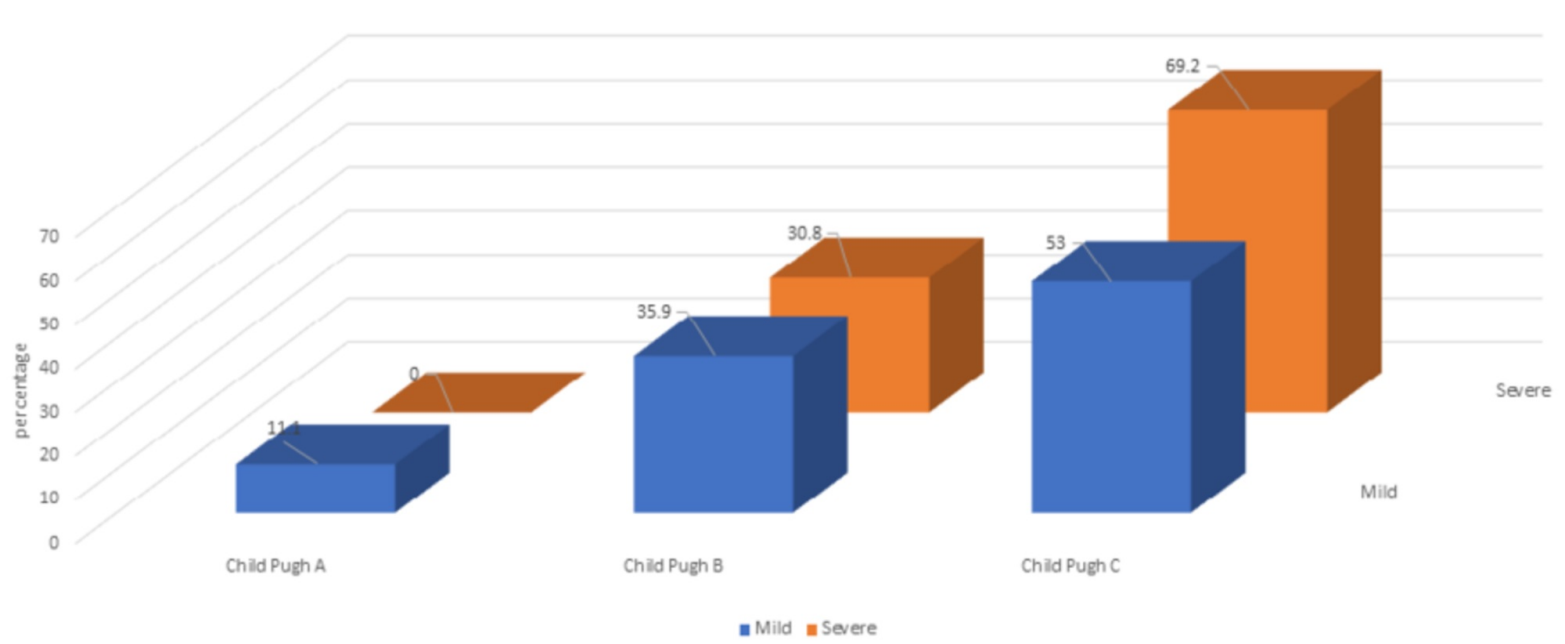
Prevalence of portal hypertensive gastropathy according to the Child-Pugh class

In a subgroup analysis of patients with PHG (n = 269), mild PHG was present in 18 (11.1%), 90 (35.9%), and 109 (53.0%) in Child-Pugh class A, B, and C, respectively. Severe PHG was present in none in Child-Pugh A, 16 (30.8%) and 36 (69.2%) in Child-Pugh B and C, respectively (Figure 3).

There were significant associations between Child-Pugh class and PHG & Child-Pugh class and PHG severity, *p *= 0.001 & *p *= 0.01 (*p* <0.05), respectively. However, there was a non-significant association between the MELD score groups and PHG.

Factors related to PHG: etiology of CLD

The cause of CLD was alcohol in 361 patients (89.4%), chronic HBV infection in 28 (6.9%), chronic HCV infection in 7 (1.7%), and NASH in 8 (2%), as shown in figure 4. No association was found between the etiology of cirrhosis and the severity of PHG (p=0.56) as shown in table 6.

**Table 6:** Distribution of portal hypertensive gastropathy according to etiology PHG, portal hypertensive gastropathy; ALD, alcoholic liver disease; HBV, hepatitis B; HCV: hepatitis C; NASH: non-alcoholic steatohepatitis

Etiology	No PHG	PHG	TOTAL
ALD	120 (29.7)	241 (59.7)	361 (89.4)
HBV	9 (2.2)	19 (4.7)	28 (6.9)
HCV	4 (1)	3 (0.7)	7 (1.7)
NASH	2 (0.5)	6 (1.5)	8 (2)
TOTAL	135 (33.4)	269 (66.6)	404

## Discussion

PHG can present at any age, including pediatric or adult age group. In our study, PHG was present in 66.6 %. There is much variance in the prevalence of PHG in the literature reported between 16% to 100% in patients with cirrhosis [10]. Of a total of 404 patients studied, mild PHG was present in 217 (53.7%) patients and severe PHG in 52 (12.9%) patients. In the study by Kumar et al., PHG was present in 55% [17]. Similarly, in another study by Gupta et al., PHG was present in 61 % of which mild PHG was present in 85% and severe in 15% of the patients [18].

There is no consistent report on the relationship of degree of portal hypertension (PHTN) with PHG. Several studies were carried out to see the association of PHG with PHTN and EV [17,19-20]. Such studies including Parikh et al., Kumar et al., Bayraktar et al., Pan et al., and Primignani et al. showed the presence and severity of PHG to correlate with the grade of varices significantly [17,21-24]. On the contrary, Gupta et al., Dong et al., Iwao et al., and Yang et al. did not find any relationship between PHG and the grade of varices [18,25-27].

In our study, EV was present in 89.6% of CLD patients. Our study also did not find a significant association between the presence and size of EV and the presence and severity of PHG. (*p* = 0.364). However, there were significant associations between Child-Pugh class and PHG and Child-Pugh class and PHG severity, *p *= 0.001 & *p *= 0.01 (*p* <0.05), respectively. Numerous studies have also given similar results. However, the reported strength of this correlation is variable. Some studies showed a correlation between all stages of cirrhosis and PHG, whereas other studies showed a correlation only for specific stages of cirrhosis. Sarin et al. reported an 87% prevalence of PHG in patients with Child-Pugh C, versus only 13% prevalence in patients with Child-Pugh A [28]. Another study reported that only Child-Pugh C was independently associated with PHG (OR = 2.68; 95%CI: 1.16-6.20, *P* = 0.021) [17].

Few studies have also incorporated the MELD score in their assessment of the severity of portal hypertension in CLD. Ahmed et al. showed MELD score >12 significantly associated with severe PHG [29]. Likewise, a similar result was shown by Kim et al. as well [19]. However, in our study, there was a non-significant association between MELD score and PHG. This could be due to limitations of the MELD scoring system itself.

Several series have reported the frequency and severity of PHG concerning different etiologies of CLD. Iwao et al., Kim et al., and Gupta et al. did not find a correlation between CLD etiology and severity of PHG in their prospective studies [18-19,26]. In our study, alcohol was the predominant cause of the CLD (89.4%) followed by chronic HBV infection (6.9%), NASH (2%) and chronic HCV infection (1.7%). Similar to other studies, we could not find any association between the etiology of cirrhosis and the severity of PHG (*p *= 0.56).

The variations in the results of the studies could be due to several factors. First, PHG is an objective diagnosis made during EGD, and so there is interobserver variation. Moreover, several classifications exist for stratifying the severity of PHG, and different researchers have used different classification system, and similar reason holds of EV as well. Most of the studies have included heterogeneous groups of population of CLD, and others have included patients of non-cirrhotic portal hypertension as well. The severity of PHG has been also associated with the duration of disease and is also related to the use of beta-blockers, or variceal ligation [17-18].

Strengths and weaknesses of the study

Limitations of this study include single-center, heterogeneous groups of patients, no liver biopsy to diagnose liver cirrhosis and no gastric mucosal biopsy to rule out the possibility of coexistence of H. pylori infection-related mucosal changes. On the other hand, the strength being the first study conducted in Nepalese CLD patients to determine the prevalence of PHG is noteworthy.

## Conclusions

While PHG is usually asymptomatic and discovered on upper endoscopy, its prevalence is 66.6 % in Nepalese patients with cirrhotics. PHG is significantly associated with severity of Child-Pugh class but not associated with MELD score and other biochemical parameters. The severity of PHG was also not related to the grade or size of EV.
